# Multi-Scale DenseNets-Based Aircraft Detection from Remote Sensing Images

**DOI:** 10.3390/s19235270

**Published:** 2019-11-29

**Authors:** Yantian Wang, Haifeng Li, Peng Jia, Guo Zhang, Taoyang Wang, Xiaoyun Hao

**Affiliations:** 1 State Key Laboratory of Information Engineering in Surveying, Mapping and Remote Sensing, Wuhan University, Wuhan 430079, China; wytcsuch@whu.edu.cn; 2 School of Geosciences and Info-physics, Central South University, Changsha 410083, China; lihaifeng@csu.edu.cn; 3 China Satellite Navigation Office, Beijing 100034, China; jiap@beidou.gov.cn; 4 School of Remote Sensing and Information Engineering, Wuhan University, Wuhan 430079, China; wangtaoyang@whu.edu.cn; 5 Shandong Aerospace Electronic Technology Institute, Yantai 264000, China; hxy_2018@163.com

**Keywords:** remote sensing images, aircraft detection, compact multi-scale dense convolutional neural network, multi-scale training

## Abstract

Deep learning-based aircraft detection methods have been increasingly implemented in recent years. However, due to the multi-resolution imaging modes, aircrafts in different images show very wide diversity on size, view and other visual features, which brings great challenges to detection. Although standard deep convolution neural networks (DCNN) can extract rich semantic features, they destroy the bottom-level location information. The features of small targets may also be submerged by redundant top-level features, resulting in poor detection. To address these problems, we proposed a compact multi-scale dense convolutional neural network (MS-DenseNet) for aircraft detection in remote sensing images. Herein, DenseNet was utilized for feature extraction, which enhances the propagation and reuse of the bottom-level high-resolution features. Subsequently, we combined feature pyramid network (FPN) with DenseNet to form a MS-DenseNet for learning multi-scale features, especially features of small objects. Finally, by compressing some of the unnecessary convolution layers of each dense block, we designed three new compact architectures: MS-DenseNet-41, MS-DenseNet-65, and MS-DenseNet-77. Comparative experiments showed that the compact MS-DenseNet-65 obtained a noticeable improvement in detecting small aircrafts and achieved state-of-the-art performance with a recall of 94% and an F1-score of 92.7% and cost less computational time. Furthermore, the experimental results on robustness of UCAS-AOD and RSOD datasets also indicate the good transferability of our method.

## 1. Introduction

With the rapid development of sensors and satellite-based remote sensing technologies, the resolution of remote sensing images has greatly improved. High-resolution images contain more abundant textural details and target information, which are conducive to the identification of various objects. As a typical civil and military target, the aircraft plays an important role in many fields, such as transportation services, wartime strikes, and air surveillance, so it is of great importance to detect aircrafts in remote sensing images.

To date, various aircraft detection methods have been proposed, which can be mainly represented by the template matching-based method [1,2], the segmentation and edged-based method [3], and the machine learning-based method [4,5]. The template matching-based method is one of the earliest proposed algorithms, which measures the similarity between template and target to obtain detection. This method extremely depends on the design of the template and can only detect the aircraft target which is consistent with the shape, size and direction of the template. The segmentation and edged-based methods focus on the obvious contour, line and edge features of the targets, which is fast and simple but susceptible to external interference. The machine learning-based method regards object detection as a process of feature extraction and classification, which firstly extracts the texture, shape and spatial relationship features by bag-of-words (BoW) [5], histogram of oriented gradients (HOG) [6], etc., and then sends features to classifiers for further determination, such as support vector machine (SVM), AdaBoost. In summary, all of these methods utilize the low-level features manually designed to describe the structures of objects, which are highly dependent on prior knowledge and have poor generalizability. They are not sufficiently robust to be applied into background-complex remote sensing images for automatic aircraft detection.

The early-stage convolutional neural network (CNN) [7] has many limitations in application due to its weak expression ability. In recent years, the development of deep learning and high-performance computing devices makes the realization of deep CNN (DCNN) possible. By constructing a multilayered neural network to simulate the organizational structure of the human cerebral cortex for perceiving external information, DCNN can automatically learn feature representations, which realizes the abstraction and description of objects hierarchically [8]. In 2014, the success of GoogleNet [9] and VGGNet [10] in the ImageNet Large-Scale Visual Recognition Challenge (ILSVRC) competition firstly introduced the DCNN publicly. VGGNet achieves the maximum depth of 19 layers by repeatedly stacking 3 × 3 convolution layers and 2 × 2 pooling layers. GoogleNet not only increases the depth of the network but also the width by parallelly performing multiple convolution and pool operations at each layer, which enhances the feature representation. However, the vanishing-gradient problem [11,12] has always been a difficulty in training DCNNs. In 2015, deep residual network (ResNet) proposed by He et al. [13] greatly alleviated this problem. ResNet involves identity mapping directly from the output of the shallow network to the deep layer through shortcut connections, which transforms the network for optimizing the residual mapping, thus reducing the difficulty of network learning. Inspired by identity mapping, Huang et al. [14] proposed DenseNet in 2017: it enhances feature propagation by dense connections and greatly reduces the number of parameters while ensuring a high performance.

Due to its powerful learning ability, DCNN has achieved top performance in the field of scene classification [15,16,17] and object detection [18,19,20]. Generally, CNN-based methods can be divided into two aspects: the two-stage detector and the one-stage detector. In 2014, the emergence of the region-based CNN (R-CNN) [21] successfully introduced deep learning into the object detection. As the ground-breaking work of two-stage algorithm, R-CNN applies the CNN to extract the features of region proposals obtained by selective search (SS) [22], which greatly improves the detection accuracy compared with traditional machine learning-based methods. Aiming for a lower time-consuming, Fast R-CNN [23] extracts the features on the whole image and maps the region proposals directly to the last convolution layers, avoiding the repeated feature extraction operations. However, an SS algorithm to generate proposals has always been a crucial factor that leads to low efficiency. In 2016, the Faster R-CNN proposed by Ren [24] replaces SS with the Region Proposal Network (RPN) and unifies feature extraction, RPN, object classification and bounding boxes regression into an end-to-end framework, which makes the object detection process more concise and obtain a significant progress in both accuracy and efficiency. Based on Faster RCNN, more excellent algorithms emerge, such as FPN [25], Mask R-CNN [26] and Cascade R-CNN [27], which greatly promotes the development of two-stage detectors. Conversely, the one-stage detector directly predicts the location and corresponding category probability of the target in the image through the regression method, e.g., You Only Look Once (YOLO) series [28,29,30]. Abandoning the process of proposal generations, YOLO acquires a real-time detection speed at the expense of accuracy. Single Shot MultiBox Detector (SSD) [31] not only absorbs the anchor mechanism of Faster RCNN and the regression idea of YOLO, but also employs the feature maps of different resolution to predict, which improves the detection accuracy and speed simultaneously. In 2018, RetinaNet [32] imports focal loss to the one-stage detector, which further alleviates the imbalance problems between positive and negative samples, outperforming all the other existing detectors.

At present, many research teams have applied the CNN-based method to detect aircrafts in high-resolution images: Xie et al. [33] proposed a robust method for tiny and dense aircraft detection by combining Region-based Fully Convolutional Networks (R-FCN) [34] and ResNet-101. By replacing standard convolution with deformable convolution, Ren et al. [35] proposed a Deformable ResNet-based Faster R-CNN method which produces a single high-level feature maps for prediction, demonstrating the effectiveness in modeling geometric variations. Guo et al. [36] adopted VGGNet into the Faster-RCNN and constructed a multi-scale base network, with the consideration of feature maps with various receptive fields. Zhang et al. [37] applied ResNet-101 as feature extraction network and introduced Online Hard Example Mining (OHEM) [38] to improve the performance of Faster R-CNN; motivated by the SSD and YOLO, Zhuang et al. [39] designed a single shot detection framework with the combination of multi-scale feature fusion and soft-Non Maximum Suppression (soft-NMS), which obtains a good tradeoff between detection accuracy and computational efficiency. Zheng et al. [40] borrowed the idea of dense connection and built a new structure called Dense-YOLO by replacing the two residual network modules in YOLO V3 [30] with two dense network modules, achieving a good performance in over-exposure, and cloud-occlusions scenes; Guo et al. [41] also applied DenseNet to SSD and designed a series of candidate boxes with different aspect ratios to detect aircraft targets of different scales. As can be seen, Faster RCNN is still the mainstream two-stage algorithm applied in aircraft target detection. Meanwhile, with the increasing demand for detection speed, one-stage detectors are gradually being widely used. However, all the methods above either improve the one-stage algorithm such as YOLO and SSD or simply use the ResNets and VGGNet as backbone, which does not explore the application of DenseNets into Faster RCNN.

Aircraft target detection in remote sensing images is sensitive to the resolution. The shapes of the same aircraft are multi-scale in different resolution images and the sizes of different types of aircraft also vary greatly in the same resolution image. Therefore, it is necessary to consider the variance of aircrafts’ scale. Additionally, the size of common types of aircrafts (e.g., F-16, F-22, etc.) is generally less than 50 × 50 pixels. After feature extraction of the DCNN, the size of aircrafts in the top-level feature map is only 1/32 of the original, approximately 1 × 1 pixel, which causes serious loss of semantic information, thus making detection very difficult. Actually, the features of each layer are the mapping of the targets on various scales that contain different semantic meanings. Prediction with only top-level features does not completely account for the contribution and difference of multi-scale features in target expression.

To mitigate the above problems, a multi-scale DenseNets-based method is proposed in this paper. Our contributions are listed as follows:

(1) We introduced DenseNet as backbone and then constructed a MS-DenseNet with the application of FPN [25], which not only enhances the propagation of features but also comprehensively utilizes both bottom-level high-resolution features and top-level semantic strong features. Additionally, we applied a multi-scale region proposal network (MS-RPN), which can produce multi-scale proposals to be responsible for targets of corresponding scale, ensuring the effectiveness for detecting small aircrafts.
(2) We developed a new compact structure named MS-Densenet-65, which effectively improves the performance of small aircrafts detection, while costing less time in both training and testing. By eliminating some unrequired convolution layers, the Densenet-65 reduces the destruction of the bottom-level high-resolution features and protects the information of small aircraft targets, which are easily submerged by redundant features.
(3) We proposed a multi-scale training strategy and design a suitable testing scale of image in detection, which allows the network to learn aircraft targets at different scales and resolutions, thus improving the robustness and generalization ability of proposed model.

The rest of this paper is organized as follows. Section 2 presents the background of DenseNet and the details of our proposed method. Section 3 presents a description of the dataset, experimental settings, and detection performance. Section 4 analyzes the results of the proposed method. Finally, Section 5 concludes this paper.

## 2. Materials and Methods

### 2.1. Dense Convolutional Network

**Figure 1:**
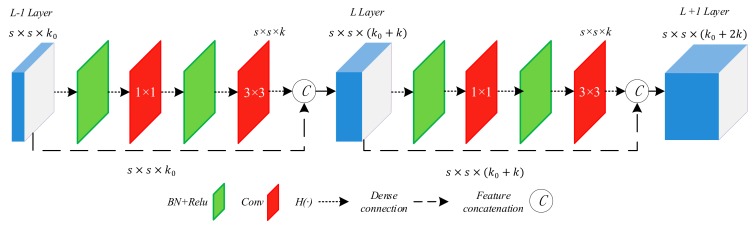
Structure of dense block. There are two dense connections in this dense block. ”BN + Relu” represents Batch Normalization and the Rectified linear unit activation function. “Conv” represents the convolution layer. The short dashed arrow represents nonlinear transformation *H*(·). The long dashed arrow represents dense connection.

The basic unit of DenseNet is a dense block, as shown in Figure 1. We denote the feature maps of the L-1 layer as $s\times s\times k_{0}$, where *s* represents the size of feature maps and $k_{0}$ represents the number of channels. *H*(·) [14] represents a nonlinear transformation, which includes a series of operations: Batch Normalization layer (BN), Rectified linear unit (Relu) activation function, a 1 × 1 convolution layer and a 3 × 3 convolution layer, as shown by the short dashed arrow. The 1 × 1 convolution operation is adopted to reduce the number of channels, aiming to improve the computational efficiency. The 3 × 3 convolution operation is used for feature reorganization. The non-linear transformation *H*(·) does not change the size of feature maps but only changes the number of channels to *k* (*k* = *32*). The long dashed arrow represents the dense connection, which directly connects the feature maps of L-1 layer to the L layer and then make a concatenation with the output of *H*(·), thus resulting in $s\times s\times (k_{0}+k)$. Similarly, the output of L+1 layer is $s\times s\times (k_{0}+2k)$.

**Figure 2:**
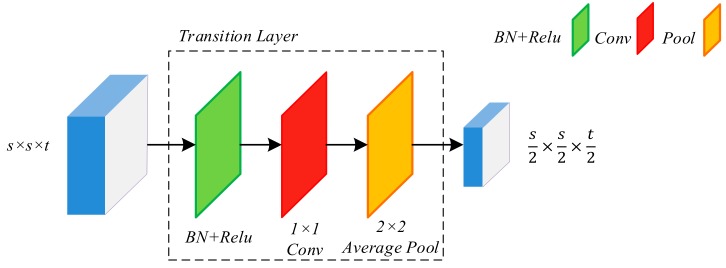
Structure of transition layer. s represents the size of feature maps and t represents the number of channels. “BN + Relu” represents Batch Normalization and the Rectified linear unit activation function. “Conv” represents the convolution layer. “Pool” represents the average pooling.

Since the number of feature maps will increase dramatically after multiple dense connections, a transition layer is designed to reduce feature dimension from previous dense block, as shown in Figure 2. The transition layer consists of BN and a 1 × 1 convolutional layer followed by a 2 × 2 average pooling layer. The 1 × 1 convolution reduces the number of channels to half of that of the previous layers while the 2 ×2 average pooling reduces the size of the feature maps.

### 2.2. Proposed Method

#### 2.2.1. Compact-DenseNets

As can be seen from in Figure 1, in DenseNet, the feature maps of all the preceding layers are used as input in the next layer, which alleviates the problem of a lack of target’s location information for the top-level features to some extent. However, in DCNN, the bottom-level features are seriously destroyed due to a large number of repeated convolution layers. In this case, DenseNet cannot propagate the bottom-level features effectively but reuse many unnecessary redundant features, making the residual sparse bottom-level features submerged by top-level features. Thus, we believe that the application of deep DenseNets in aircraft target detection, especially in small aircraft targets detection, is not an optimal method.

Thus, in this paper, by compressing some repeated dense connections in each dense block, we constructed three Compact-DenseNets, which allow less feature propagations compared with deep DenseNets. On the one hand, Compact-DenseNets reduce the destruction of the bottom-level features, on the other hand, they do not generate more redundant features, which protects the features of small targets from being submerged and meanwhile improves the efficiency of training and testing. The structures of the Compact-DenseNets, which are called DenseNet-41, DenseNet-65, and DenseNet-77, respectively, according to the number of layers, are shown in Table 1.

#### 2.2.2. DenseNet-based Feature Pyramid Networks

**Figure 3:**
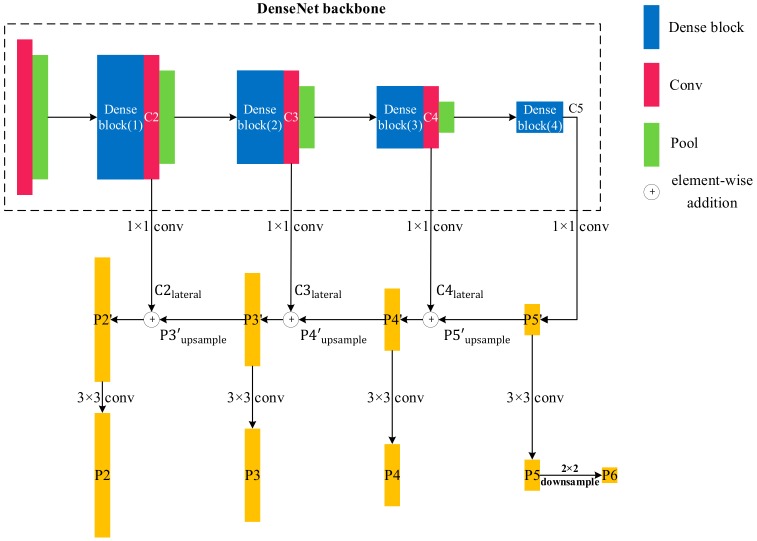
DenseNet-based feature pyramid network. $Ci_{lateral}$ represents output of lateral connection. $Pi\prime_{upsamlpe}$ represents output of upsampling. C5 represents the output of the dense block (4).

Herein, we combine FPN with DenseNets instead of the conventional ResNets. The architecture of the MS-DenseNet is shown in Figure 3. We select the output of the 1 × 1 convolution layer of each transition layers as the salient feature maps, namely {Conv_2, Conv_3, Conv_4}, as shown in Table 1. This was performed because these layers are the recombination and integration of the features extracted from the previous dense block, which not only possess the strongest expressive ability but also reduce the number of channels. For an easier expression, we abbreviate them to {C2, C3, C4}. Since there is no transition layer after the dense block (4), we directly select the output of dense block (4) as the salient feature map, denoted as C5. Compared to the input image, {C2, C3, C4, C5} have strides of {4, 8, 16, 32} pixels, and exhibit scale diversity. The steps for establishing the FPN with {C2, C3, C4, C5} are as follows:

1. P5′ is generated from C5 through a 1 × 1 convolution layer, which is called lateral connection. This operation reduces the number of feature map channels to 256 and recombines features simultaneously. The expression for this operation is:
  (1) $$C5\overset{1\times 1\times 256\;conv\;(lateral\;connection)}{\to }P5\prime ;$$
2. Semantically coarser but higher-resolution feature maps $P5\prime_{upsample}$ are generated from P5′ via nearest upsampling with a step size of 2. Meanwhile, $C4_{lateral}$ is generated from C4 via lateral connections:
  (2) $$P5\prime \overset{2\;\times \;upsample}{\to }P5\prime_{upsample};$$
  (3) $$C4\overset{1\times 1\times 256\;conv\;(lateral\;connection)}{\to }C4_{lateral};$$
3. Since $C4_{lateral}$ has the same size as $P5\prime_{upsample}$, we fuse them by element-wise addition:
  (4) $$C4_{lateral}+P5\prime_{upsample}=P4\prime ;$$
4. Using the same operation as step 2–3, P3′ and P2′ are successively generated.
5. To eliminate the aliasing effect caused by upsampling, a 3 × 3 convolution layer is adopted on {P2′, P3′, P4′, P5′}, resulting in {P2, P3, P4, P5}. The scale of {P2, P3, P4, P5} corresponds to the original features {C2, C3, C4, C5}. Then, P6, which has the strongest semantic information, is generated from P5´ by downsampling. The feature maps {P2, P3, P4, P5, P6} are ultimately used for prediction.

#### 2.2.3. Multi-Scale Region Proposal Network

**Figure 4:**
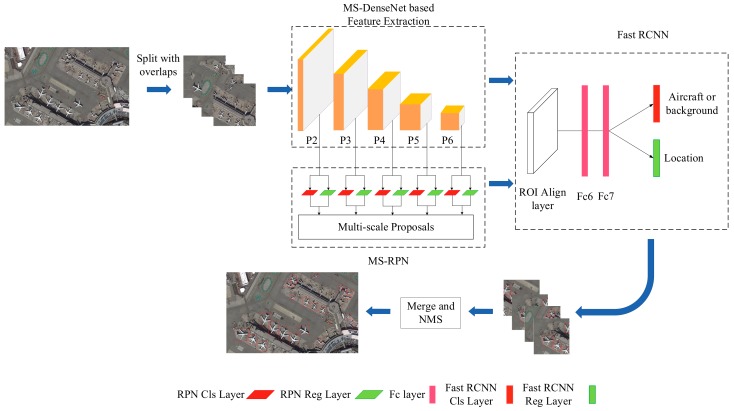
Overall framework of aircraft detection from remote sensing images. “Fc” represents the fully connected layer. “Cls” represents the proposal classification layer. “Reg” represents the proposal regression layer. “NMS” represents the non-maximum suppression algorithm. “ROI” represents the region of interest.

The conventional RPN only generates three kinds of larger anchors with areas of (128^2^, 256^2^, 512^2^) on the top-level feature maps, which is obviously not suitable for the multi-scale features. As mentioned in Section 2.2.2, {P2, P3, P4, P5, P6} are feature maps with different resolutions and spatial scales; thus, they can be utilized to generate multi-scale proposals to detect aircrafts of corresponding resolutions and scales, as shown in MS-RPN component of Figure 4 in Section 2.2.5. In other words, the bottom-level feature maps with high resolutions (e.g., the P2 layer) can be adopted for detecting small aircrafts, while the coarse resolution feature maps with strong sematic meanings (e.g., the P5 and P6 layers) can be adopted in large aircraft detection.

#### 2.2.4. Multi-Scale Training

Although deep learning provides a powerful tool for automatic object detection, the variation in aircraft sizes at various resolutions is still an important factor that affects the detection performance. The FPN proposed in Section 2.2.2 only produces multi-resolution feature maps for a size-fixed image, which is not enough for resolution-sensitive aircraft targets. Therefore, five different scales are chosen in the experiment: 768 × 768, 896 × 896, 1024 × 1024, 1156 × 1156, and 1280 × 1280. During network training, each sample is randomly scaled so that the network can learn features of different resolutions.

#### 2.2.5. Aircraft Detection Process

The overall framework of our method is shown in Figure 4. The framework mainly consists of three components: MS-DenseNet-based feature extraction, MS-RPN for generating region proposals, and Fast-RCNN for object recognition and location regression.

Remote sensing images cannot be directly sent into the network for aircraft target detection due to the wide coverage of image and the limitation of Graphic Processing Unit (GPU) computing performance. Thus, we first divide images into sub-images with the size of 1024 × 1024 pixels. The overlap between adjacent sub-images is 100 pixels, to prevent large objects from being truncated.

Sub-images are then sequentially fed into MS-DenseNet for feature extraction. Then, MS-RPN generates multi-scale candidate region proposals for the Fast-RCNN.

ROI Align layer is used to convert the multi-scale region proposals into feature maps with a fixed size of 7 × 7 pixels. Then, these feature maps are fed into box-classification layer and box-regression layer, respectively, to achieve aircraft recognition and location regression.

Differently from the conventional detection process, we do not apply NMS algorithm for each sub-image, but only once for the merged image, which improves the detection efficiency and also removes targets in overlapping regions.

## 3. Experiments and Results

### 3.1. Implementation Details

#### 3.1.1. Experimental Dataset

The experimental data in this study are high-resolution image DOTA dataset [42], which are collected from Google Earth and JL-1 satellite. The dataset includes 244 scenes of images with various resolutions ranging from 0.1 to 1 m, containing the conditions of high-density objects, and complex backgrounds. Using a ratio of 7:3, 171 images were randomly selected as the training set and the remaining 73 samples served as the test set. We divided training images into 1024 × 1024-pixel sub-images with an overlap of 100 pixels, and then data cleaning was used to filter out sub-images without objects, resulting in 1696 training samples. Finally, a rotation method (90°, 180°, 270°) was adopted during data augmentation, which resulted in 3733 samples for training, including aircraft targets with different orientations.

**Figure 5:**
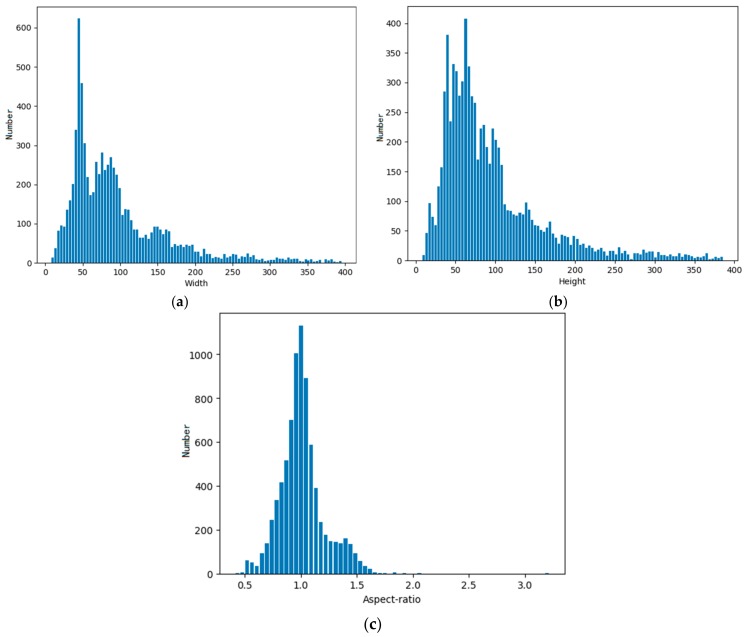
Distribution of aircraft shapes in the training set: (**a**) width, (**b**) height, and (**c**) aspect ratio.

Moreover, we also statistically analyzed aircraft distributions of the training set, as shown in Figure 5. It is obvious that the number of aircrafts with side length ranging from 20 to 100 pixels occupy an especially large proportion, and most of aircrafts are around 50 pixels in length and width. As per the statistics, we divide the test set into small, medium, and large targets, as shown in Table 2. The targets whose area is less than 70² pixels are recorded as small targets, and those whose area is larger than 150² pixels are recorded as large targets. The remaining targets are medium targets. It can be seen that the number of small targets in the test set accounts for about 50% of the total.

#### 3.1.2. Anchor Settings

From Figure 5, it can be also seen that the length and width of most aircrafts are within 300 pixels, and the distribution of aspect ratios is mainly between 0.5 and 2. Thus, we define the anchors with the areas of {16^2^, 32^2^, 64^2^, 128^2^, 256^2^} pixels on {P2, P3, P4, P5, P6} respectively, and three multiple-aspect ratios {1:2, 1:1, 2:1} are applied to each level. Thus, 15 types of anchors are generated in MS-RPN.

#### 3.1.3. Parameter Setting

There are two training strategies for Faster-RCNN. One is alternating optimization, which trains the RPN and Fast-RCNN alternately. The other is end-to-end training, in which the RPN and Fast-RCNN are trained simultaneously. The second strategy can achieve the same training effect as the first strategy, while also greatly improving the training efficiency. Therefore, we used end-to-end training in our method. We trained a total of 70,000 iterations with the momentum of 0.9 and a weight decay of 0.0001. The basic learning rate (*base_lr*) was set as 0.0025, with a step strategy of *gamma* = 0.1. Thus, the learning rate (*lr*) of current iteration (*iter*) could be calculated by the formula:

(5) $$lr=base\_lr\;\times \;gamma^{current\_step\;},\;\mathrm{where}$$

(6) $$current\_step=\left\{\begin{array}{ll} 0 & iter\in \left[0,39999\right]\\ 1 & iter\in \left[40000,59999\right]\\ 2 & iter\in \left[60000,69999\right] \end{array}\right.$$

In the training stage of the RPN, in order to ensure the integrity of positive samples, anchors with intersections over unions (IOUs) > 0.7 were labeled as positive samples, while anchors with IOUs < 0.3 were labeled as negative samples. The proportion of positive and negative samples was set to 1:1, totaling 256 samples. Additionally, due to the application of the FPN, 12,000 proposals were first selected for each level of the FPN, and then the top 2000 proposals with high scores were finally selected for Fast-RCNN training.

### 3.2. Evaluation Metrics

In our experiments, recall and precision were introduced to evaluate the performance of our method. They can be calculated by Equations (7) and (8), where TP (True Positive) denotes the number of positives that are identified correctly, FN (False Negative) denotes the number of positives that are misidentified as negatives, and FP (False Positive) denotes the number of negatives that are misidentified as positives. In our evaluations, we assumed a predicted box to be correct if its intersection area with the ground truth exceeded 0.5.

(7) $$Recall=\;\frac{TP}{TP+FN}$$

(8) $$Precision=\;\frac{TP}{TP+FP}$$

It can be seen from Equation (7) that the recall measures the proportion of positives that are predicted correctly, while the precision Equation (8) focuses on the proportion of true positives detections. Different confidence scores can lead to different numbers of TP, FN, and FP, thus affecting recall and precision. Generally, a lower confidence can achieve a higher recall while more negatives will be also predicted as positives, leading to a decrease in precision. Conversely, a higher confidence can improve precision, while the recall decreases. An excellent detection method usually has both higher recall and precision. Therefore, we added F1-score as another evaluation metric, which is defined as the harmonic mean of recall and precision. In the experiments, all the evaluation metrics were calculated at a threshold of 0.7.

(9) $$F1-score=\frac{2\times Recall\times Precision}{Recall+Precision}$$

### 3.3. Performance Comparison of Different Compact-DenseNets

To evaluate the performance of the three Compact-DenseNets proposed in this paper, DenseNet-121 [14] was added for comparison. Similarly to the structure of Compact-DenseNets, DenseNet-121 also contains four dense blocks and three transition layers, but possesses more convolution layers in dense blocks. All the models were trained and tested with the single scale of 1024 × 1024 on the same dataset. The experimental results are shown in Table 3.

It is can been seen that MS-DenseNet-65 has an equivalent recall and precision and obtains the best F1-Score of 91.7% among the Compact-DenseNets. Compared with the MS-DenseNet-121, MS-DenseNet-65 improved recall by 1.4%, and is more efficient with a training time of 0.156 s/iter and a testing time of 0.094 s/image. In addition, MS-DenseNet-77 also achieves a F1-Score that is equivalent to MS-DenseNet-121. MS-DenseNet-41 further reduces feature propagation in each dense block, thus consuming the least computational time. However, fewer layers also lead to a lack of features, which is not conducive to learn the differences between targets and backgrounds, thus resulting in poor recall and precision.

Table 4 reflects the recall rates of each model in detecting small, medium, and large targets. We can see that all models perform well in detecting medium and large targets. The noticeable performance difference between the models is observed during small targets’ detection. MS-DenseNet-65 obtain the highest recall rate of 86% in detecting small aircraft targets, which is 3.5% higher than that of MS-DenseNet-121. The recall rate of MS-DenseNet-77 is also 1.4% higher than that of MS-DenseNet-121. Additionally, MS-DenseNet-41 also achieves the same recall rate as MS-DenseNet-121.

**Figure 6:**
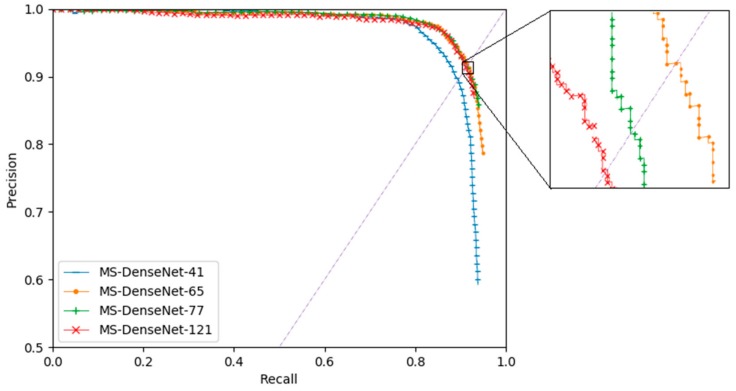
The P–R curves of different Compact-DenseNets.

Figure 6 plots the precision-recall (P–R) curves of each model. The dotted line connects point (0,0) and point (1,1). The intersection of the dotted line and the P–R curve is equilibrium point. The location of the equilibrium point can be used to measure the performance of the model. The closer it is to point (1,1), the better the performance will be. Therefore, it is apparent that MS-DenseNet-41 performs the worst while MS-DenseNet-65 performs the best.

### 3.4. Influence of Different Training and Testing Scales

As stated before, we adopted five different scales for training samples. Different scales can produce different resolution samples. Therefore, to explore the impact of different scales in aircraft detection, we performed 19 groups of experiments. All the experiments were based on MS-DenseNet-65. Groups 1–15adopted a single scale t ($t\in \left\{768,896,1024,1152,1280\right\}$) for network training. For a fixed training scale t, three scales were selected for testing, denoted as t, t + 256, and t + 512. Groups 16–19 were trained with multi-scale strategy but tested with a single scale. Table 5 summarizes the experimental results of various groups.

It is obvious that both the training and testing scale have a great impact on the detection performance. With the increase of the testing scale, the recall rate first increases and then decreases, while the precision rate continues to decrease. In the single-scale training groups, such as Groups 1–3, 4–6, 7–9, 10–12, and 13–15, when the testing scale is set to t + 256 (t denotes the training scale), the recall rate increases significantly, e.g., the recall of Group 2 is 2.5% higher than that of Group 1, and the recall of Group 5 is 2.6% higher than that of Group 4. However, due to the reduction of precision, F1-score only has a slight improvement. In the multi-scale training groups (Group 16–19), when the testing scale is set to 1024 × 1024, the MS-DenseNet-65 obtain the best performance, with a recall of 94% and an F1-Score of 92.7%.

### 3.5. Comparison with Other Methods

To evaluate the performance of MS-DenseNet-65 in aircraft target detection, a series of experiments were performed as comparisons, including ResNets-based Faster RCNN, SSD, and RetinaNet. All the selected methods were trained with the single scale and the testing scales were set to 1024 × 1024. The detection results are shown in Table 6.

It can be seen that our method achieves state-of-the-art performance: 94% for recall, 91.4% for precision, and 92.7% for F1-score, which outperforms other methods. The computational efficiency is also an important indicator for evaluation. We can see that our method ensures the best performance while keeping the training and testing relatively fast, with a training speed of 0.168 s/iter and a testing speed of 0.094 s/image, which ranks third. Therefore, considering both detection performance and computational efficiency, our method is the best choice.

The recall of each method for detecting small, medium, and large targets is shown in the Table 7. It is apparent that our method makes great progress in detecting small aircraft targets. The recall rate of our method is nearly 10% higher than that of ResNets-based Faster RCNN, 16% higher than that of RetinaNet, and 49.3 % higher than that of SSD, which illustrates the great superiority.

**Figure 7:**
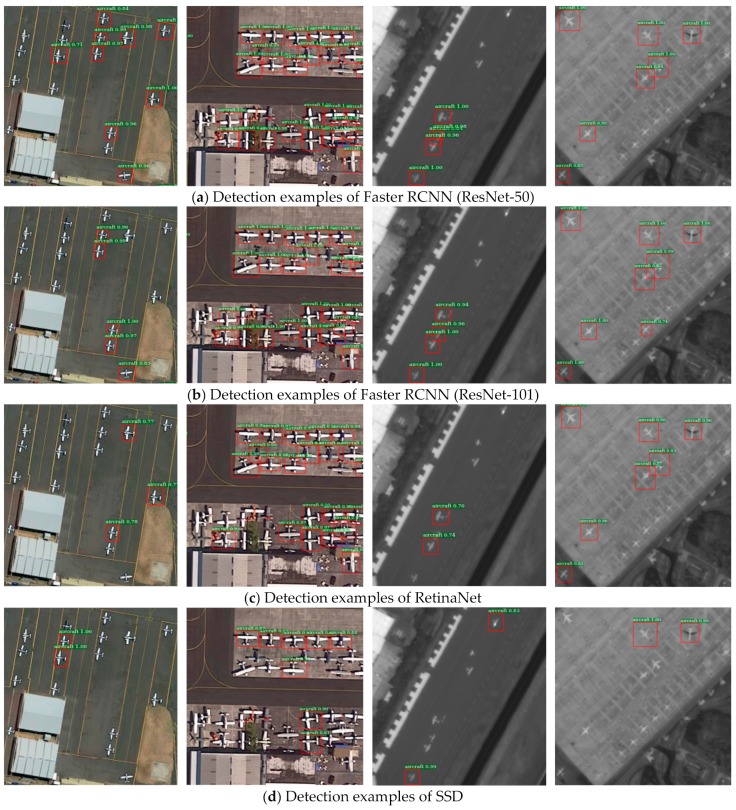

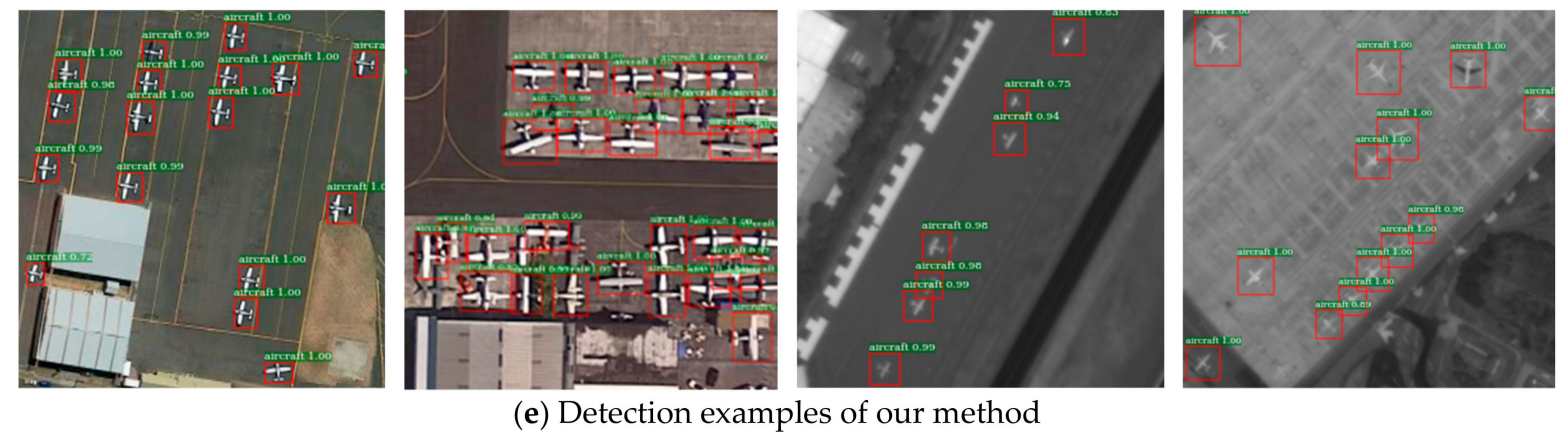
Detection examples of DOTA dataset.

To further prove the effectiveness of the proposed method, the qualitative results between our method and other four comparison methods are shown in Figure 7. The resolution of the first and second columns images is 0.27 m, while that of third and fourth columns images is 0.72 m. These images contain a large number of dense small aircraft targets and contour-blurred targets. It can be seen that our method achieves an excellent performance in detecting small aircrafts, especially in lower resolution image (third and fourth columns) detection, in which all the aircrafts are detected correctly. As a typical one-stage detector, due to the significant imbalance of positive and negative samples in training and detection, SSD has the worst detection result and many aircraft targets are missed out. RetinaNet introduces focal loss, which solves the limitation of one-stage detector to some extent, thus the detection performance is better than SSD. Compared with SSD and RetinaNet, Faster R-CNN has a better performance. However, it only utilizes the top-level coarse feature maps to predict, which is not conducive to the expression of small aircraft targets, so the performance is not as good as our method.

### 3.6. Transferability

In order to test the robustness and generalization ability of the proposed MS-DenseNet-65, two new data were selected as test datasets, namely UCAS-AOD [43] and RSOD [44]. Similarly to the DOTA dataset, we also statistically analyzed the distribution of small, medium, and large targets of the two datasets, as shown in Table 8. It is obvious that small and medium aircrafts account for a very large proportion in both two datasets, and the small aircrafts even occupies more than 70% in the RSOD dataset. Thus, it is more convincing to utilize these two datasets for robustness experiments.

**Figure 8:**
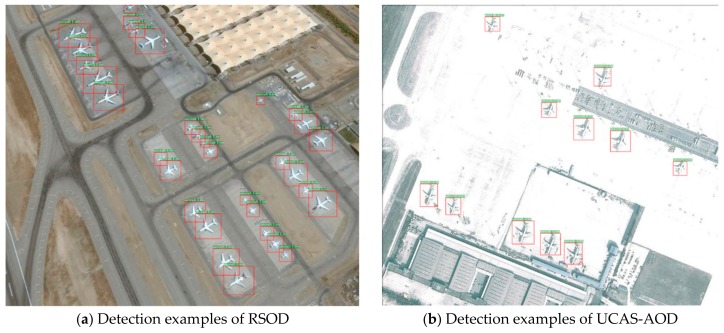
Detection examples of our method in RSOD and UCAS-AOD.

The experimental results with regards to the recall, precision, and F1-score are shown in Table 9. It can be seen that MS-DenseNet-65 still achieves a good detection performance in the new datasets, with F1-scores of 96.3% and 92.8% respectively. In addition, the recall rate of detecting small aircrafts targets also validate the effectiveness of our method, i.e., 93.4% and 88.7% from Table 9 and Figure 8 shows the detection examples on UCAS-AOD and RSOD, in which the small targets can be detected well. Moreover, we can also see that the aircraft targets under exposure conditions can still be detected, which reveals a good robustness.

## 4. Discussion

**Figure 9:**
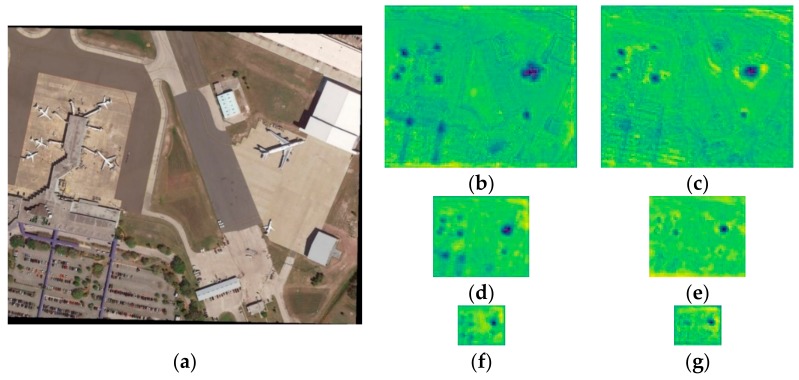
Comparison of feature maps between MS-DenseNet-65 and MS-DenseNet-121. (**a**) Input image; (**b**) P3 layer feature of MS-Densenet-65; (**c**) P3 layer feature of MS-Densenet-121; (**d**) P4 layer feature of MS-Densenet-65; (**e**) P4 layer feature of MS-Densenet-121; (**f**) P5 layer feature of MS-Densenet-65; (**g**) P5 layer feature of MS-Densenet-121;.

The quantitative analysis in Table 3 and Table 4 shows that our proposed MS-DenseNet-65 makes a great progress in detecting small aircraft targets, with a 3.5% recall improvement over MS-DenseNet-121, and maintains fast training and testing. Generally, the more layers a network has, the more expressive it will be. However, with an increase in network layers, the bottom-level features will be seriously destroyed. Figure 9b,d,f represent the feature maps of MS-DenseNet-65, while Figure 9c,e,g represent the feature maps of MS-DenseNet-121. Compared with Figure 9c the large aircraft target in Figure 9b possesses obvious contour features and the target feature is more abundant. In addition, it is clear that Figure 9d,f can still express small aircraft targets, while some of the aircraft targets in Figure 9e,g have disappeared. These results prove that the repetitive convolution layers are not all effective for small aircraft detection. Less feature propagation can also promote the performance of the network.

In Table 5, we can observe that when the testing scale is set to 1024 × 1024, the multi-scale training method achieves the best detection performance with a recall of 94% and F1-Score of 92.7%, which are far ahead of the single-scale training method. The reason is that multi-scale training improves the expression ability of the detector on different resolution aircraft targets. In addition, it is obvious that with the increase of testing scale, the recall increases first and then decreases. The reason is that enlarged remote sensing images enhance the resolution features of the small objects and make them easier to detect. However, with the further increase of the testing scale, the distribution of the large aircraft targets will be also destroyed, which leads to missing of large aircraft targets. The experimental results show that it is very important to select a suitable testing scale. For a single training scale of t, we find that when the testing scale is set to t + 256, the network achieves the best performance.

The comparative experiments of Table 6 and Table 7 reveal that our method has a great advantage in small aircrafts detection. From Figure 7, we can also see that our method is capable of detecting aircraft targets of different resolutions and shows strong feature representation ability in the detection of dense small targets. Moreover, in the test experiments of two new data sets UCAS-AOD and RSOD, our method can still obtain more than a 92% F1-score, which demonstrates a good transferability.

## 5. Conclusions

In this paper, we proposed a DenseNet-based aircraft detection method that is effective for small and multi-scale aircrafts in high-resolution remote images. Firstly, we adopted DenseNet as backbone, which enhances the reuse of the bottom-level features. Secondly, we combined FPN and DenseNet to form a MS-DenseNet with the simultaneous consideration of bottom-level high-resolution features and top-level semantically rich features. By eliminating some redundant convolution layers, a compact structure MS-DenseNet-65 was designed to protect small aircrafts features that are easily destroyed. Moreover, a multi-scale training strategy is adopted, which makes the detector more adaptable and robust on different resolution aircraft targets. The comparative experiments show that the compact MS-DenseNet-65 achieves an excellent performance, with a great improvement in detecting small aircrafts. Additionally, the experiments on two new datasets also prove the good transferability of our proposed method.

## Figures and Tables

**Table 1:** Structure of three proposed Compact-DenseNets.

Layer		DenseNet-41	DenseNet-65	DenseNet-77
Conv_1		7 × 7 conv, stride2		
Pool_1		3 × 3 max pool, stride2		
Dense block (1)		$\left[\begin{array}{cc} 1\times 1 & conv\\ 3\times 3 & conv \end{array}\right]\times 3$	$\left[\begin{array}{cc} 1\times 1 & conv\\ 3\times 3 & conv \end{array}\right]\times 6$	$\left[\begin{array}{cc} 1\times 1 & conv\\ 3\times 3 & conv \end{array}\right]\times 6$
Transition Layer	Conv_2	1 × 1 conv		
	Pool_2	2 × 2 average pool, stride2		
Dense block (2)		$\left[\begin{array}{cc} 1\times 1 & conv\\ 3\times 3 & conv \end{array}\right]\times 6$	$\left[\begin{array}{cc} 1\times 1 & conv\\ 3\times 3 & conv \end{array}\right]\times 9$	$\left[\begin{array}{cc} 1\times 1 & conv\\ 3\times 3 & conv \end{array}\right]\times 12$
Transition Layer	Conv_3	1 × 1 conv		
	Pool_3	2 × 2 average pool, stride2		
Dense block (3)		$\left[\begin{array}{cc} 1\times 1 & conv\\ 3\times 3 & conv \end{array}\right]\times 6$	$\left[\begin{array}{cc} 1\times 1 & conv\\ 3\times 3 & conv \end{array}\right]\times 9$	$\left[\begin{array}{cc} 1\times 1 & conv\\ 3\times 3 & conv \end{array}\right]\times 12$
Transition Layer	Conv_4	1 × 1 conv		
	Pool_4	2 × 2 average pool, stride2		
Dense block (4)		$\left[\begin{array}{cc} 1\times 1 & conv\\ 3\times 3 & conv \end{array}\right]\times 3$	$\left[\begin{array}{cc} 1\times 1 & conv\\ 3\times 3 & conv \end{array}\right]\times 6$	$\left[\begin{array}{cc} 1\times 1 & conv\\ 3\times 3 & conv \end{array}\right]\times 6$
Classification Layer		7 × 7 global average pool		
		Fully-connected layer		
		SoftMax		

**Table 2:** Aircraft distribution of test set.

Target	Target Amount	Percentage
Small targets	1854	48.41%
Medium targets	1209	31.56%
Large targets	767	20.03%
Total	3830	100%

**Table 3:** Comparisons of different Compact-DenseNets.

Model	Recall	Precision	F1-Score	Train Time (s/iter)	Test Time (s/image)
MS-DenseNet-41	0.897	0.89	0.893	0.120	0.089
MS-DenseNet-65	0.918	0.917	0.917	0.156	0.094
MS-DenseNet-77	0.905	0.926	0.915	0.17	0.096
MS-DenseNet-121	0.904	0.923	0.913	0.193	0.124

**Table 4:** Recall of different Compact-DenseNets in detecting small, medium, and large targets.

Model	Recall		
	Small Targets	Medium Targets	Large Targets
MS-DenseNet-41	0.825	0.970	0.973
MS-DenseNet-65	0.860	0.972	0.966
MS-DenseNet-77	0.839	0.969	0.964
MS-DenseNet-121	0.825	0.974	0.973

**Table 5:** Detection performance of MS-DenseNet-65 with different train and test scales.

Group	Train Scale	Test Scale	Recall	Precision	F1-Score	Test Time (s/image)
1	768	768	0.907	0.909	0.908	0.077
2		1024	0.932	0.897	0.912	0.094
3		1280	0.898	0.877	0.887	0.128
4	896	896	0.907	0.909	0.908	0.088
5		1152	0.933	0.906	0.919	0.115
6		1408	0.918	0.890	0.904	0.147
7	1024	1024	0.918	0.917	0.917	0.095
8		1280	0.926	0.907	0.916	0.126
9		1536	0.919	0.891	0.905	0.179
10	1152	1152	0.916	0.920	0.918	0.110
11		1408	0.934	0.913	0.923	0.145
12		1664	0.925	0.901	0.913	0.204
13	1280	1280	0.919	0.923	0.921	0.129
14		1536	0.935	0.914	0.924	0.178
15		1792	0.927	0.908	0.917	0.238
16	768,896,1024,1152,1280	768	0.911	0.923	0.917	0.076
17		1024	0.940	0.914	0.927	0.094
18		1280	0.940	0.903	0.921	0.127
19		1536	0.930	0.888	0.908	0.178

**Table 6:** Comparison of aircraft detection results with different methods.

Method	Backbone	Recall	Precision	F1-Score	Train Time (s/iter)	Test Time (s/image)
Faster RCNN	ResNet-50	0.890	0.946	0.917	0.545	0.111
	ResNet-101	0.886	0.948	0.916	0.683	0.134
RetinaNet	ResNet-50	0.860	0.920	0.889	0.142	0.086
SSD	VGGNet-16	0.650	0.943	0.774	0.102	0.025
Ours	MS-DenseNet-65	0.940	0.914	0.927	0.168	0.094

**Table 7:** Recall of different method in detecting small, medium, and large targets.

Method	Backbone	Recall		
		Small Targets	Medium Targets	Large Targets
Faster RCNN	ResNet-50	0.799	0.978	0.974
	ResNet-101	0.791	0.978	0.971
RetinaNet	ResNet-50	0.738	0.986	0.957
SSD	VGGNet-16	0.405	0.891	0.943
Ours	MS-DenseNet-65	0.898	0.982	0.973

**Table 8:** Target distribution of UCAS-AOD and RSOD.

Target	UCAS-AOD		RSOD	
	Target Amount	Percentage	Target Amount	Percentage
Small targets	2531	33.83%	4148	77.19%
Medium targets	4402	58.83%	1226	22.81%
Large targets	549	7.34%	0	0
Total	7482	100%	5374	100%

**Table 9:** Detection results of UCAS-AOD and RSOD.

Dataset	Recall			Precision	F1-Score
	Small Targets	Medium Targets	Large Targets		
UCAS-AOD	0.934	0.987	0.990	0.957	0.963
RSOD	0.887	0.974	−	0.948	0.928
